# Lexical priming of function words and content words with children who do, and do not, stutter

**DOI:** 10.1016/j.jcomdis.2008.01.004

**Published:** 2008-11

**Authors:** Ceri Savage, Peter Howell

**Affiliations:** a The Institute of Psychiatry, Kings College London, United Kingdom; b Department of Psychology, University College London, Gower Street, London WC1E 6BT, United Kingdom

## Abstract

The specific mechanisms that underlie childhood stuttering are not fully understood. The current study investigated these mechanisms by comparing the effect on fluency of priming different components of a short sentence. The main findings were that: (1) both children who stutter (CWS) (*n* = 12, M age = 6;3) and children who do not stutter (CWNS) (*n* = 12, M age = 6;6) were more fluent after function word (FW) priming than content word (CW) priming, (2) this effect was significantly greater for CWS than for CWNS, and (3) after FW priming, CWS produced CWs with significantly longer duration than did CWNS. These findings are discussed in relation to two competing theories of stuttering: the covert repair hypothesis (CRH) [Kolk, H., & Postma, A. (1997). Stuttering as a covert repair phenomenon. In R. F. Curlee & G. M. Siegel (Eds.), *Nature and treatments of stuttering: New directions* (pp. 182–203). Needham Heights, MA: Allyn & Bacon] and the developmentally focused model of Howell and Au-Yeung [Howell, P., & Au-Yeung, J. (2002). The EXPLAN theory of fluency control and the diagnosis of stuttering. In E. Fava (Ed.), *Current issues in linguistic theory series: Pathology and therapy of speech disorders* (pp. 75–94). Amsterdam: John Benjamins].

**Learning outcomes**: After reading this article, the reader will be able to: (1) understand which linguistic levels can be primed in children who stutter; (2) see why EXPLAN predicts asymmetrical effects on fluency when function or content words are primed; (3) appreciate the distinguishing characteristics of CRH and EXPLAN theories.

Stuttering is a developmental problem. It can both inform and be informed by studies of development, and language development in particular. About 80% of children who stutter (CWS) spontaneously recover by adulthood (Andrews et al., 1983; Andrews & Harris, 1964; Yairi & Ambrose, 1999). Does the way CWS generate speech change as they grow older or are the speech generation processes of speakers who persist in the disorder and those who recover, qualitatively different from the start? To answer this question, it is necessary to identify the mechanisms that underlie the generation of fluent and disfluent speech in connected discourse, and how they are related. This is a central question in stuttering research and there is no consensus about the answer. There is disagreement in the literature over whether one mechanism results in all forms of disfluent speech (Au-Yeung & Howell, 2002), or whether ‘normal disfluencies’ and ‘stuttered speech’ are distinct forms which potentially are the result of different mechanisms (Onslow & Packman, 2005). The current paper aims to contribute to our knowledge of the processes behind stuttering by specifying the nature of the speech production mechanisms that underpin fluent and disfluent speech in both CWS and fluent children.

## 1 Existing models of the mechanisms that underlie disfluent speech

Kolk and Postma's (1997) covert repair hypothesis (CRH) proposes that all disfluent speech is caused by ‘covert repairs’ of phonological encoding errors that speakers detect before they are expressed overtly (Levelt, 1989; Levelt, Roelofs, & Meyer, 1999). In the example “*Turn left at the, no, turn right at the crossroads*”, the word “left” is selected in error, this is detected by the auditory system when it is spoken out loud and the speaker makes the correction. According to CRH, errors can be detected at the phonological level before they are spoken, resulting in covert interruption and repair. The earlier example might then be realized as “*Turn, turn right at the crossroads*”. The wrong word is produced because words related to the one intended are activated concurrently and can be selected (Kolk & Postma, 1997). A covert repair results when the erroneous word is detected and corrected internally rather than externally.

The CRH suggests that children who recover from stuttering by adulthood are similar to fluent speakers. Those whose stuttering persists represent the ‘true’ stuttering population whose speech processing is distinct from that of fluent speakers. They produce more disfluencies because their phonological processing is slower than that of fluent speakers, leading them to select a word before the activation has resolved more often, and so need to make more covert repairs. The evidence for such a difference is limited, as the CRH was developed from adult data. Given that most CWS recover, adults who stutter represent only a small proportion of those who stuttered as children, so findings cannot be generalised from one age group to another. Yaruss and Conture (1996) applied the CRH to children, They reasoned that if speech disfluencies reflect covert repairs to underlying errors, then they should be subject to the same influences that give rise to overt repairs. Consistent with this, speech errors and disfluencies were correlated in the naturalistic data of 3–6-year-old CWS. However, covert repairs are not the only possible explanation for the data and the study is as yet an isolated finding. The CRH requires more developmental evidence and it remains an open question as to whether it can account for childhood stuttering.

An alternative theory about stuttering is the EXPLAN model (derived from ‘EX’, the speech execution mechanism, and ‘PLAN’, the parallel language planning mechanism). The development, and evidence in favour, of EXPLAN are reviewed by Howell (2002, 2004) and Howell and Au-Yeung (2002). These authors argue that language planning and execution are parallel independent processes with neither process being monitored for errors. Consequently, they reject the notion of covert repairs, and instead propose that disfluent speech reflects a mismatch between the timing of planning and the timing of execution. Specifically, whilst someone is executing speech, they can plan upcoming speech. Disfluency occurs if a speaker speaks fast and finishes executing one segment before the plan for the next segment is ready. Consistent with this view, evidence has been published that shows planning difficulty (Dworzynski & Howell, 2004; Howell, Au-Yeung, Yaruss, & Eldridge, 2006) and local increases in speech rate (Howell, Au-Yeung, & Pilgrim, 1999; Howell & Sackin, 2000) affect fluency. Like the CRH, EXPLAN explains the disfluencies of fluent speakers and people who stutter (PWS) in the same way. Unlike the CRH, EXPLAN suggests that the processing mechanism of PWS starts out the same as those of fluent speakers. In childhood, the immature speech planning system is often not ready for an upcoming word. Children can respond in two alternate ways, ‘stalling’ or ‘advancing’. They may stall the upcoming speech by pausing or repeating the preceding function word (FW), for which the plan remains available. Function words are pronouns, articles, prepositions, conjunctions and auxiliary verbs (Hartmann & Stork, 1972; Quirk, Greenbaum, Leech, & Svartvik, 1985) which are phonologically simple. Alternatively, they may advance to the next word before the plan is complete which is usually a content word (CW) and thus need to repeat or prolong parts of the word whose plan is not ready until planning is complete. Content words of the grammatical classes nouns, main verbs, adverbs and adjectives (Hartmann & Stork, 1972; Quirk et al., 1985) which are phonologically complex.

EXPLAN suggests that young CWS do not differ from fluent speakers, except that they represent the slower end of the normal continuum for speech planning. The conception in EXPLAN is different from the CRH, because the planning difference is global and not specifically phonological. Most speakers recover as their speech processing system matures, which accounts for the high spontaneous recovery rates for childhood stuttering (Andrews et al., 1983; Andrews & Harris, 1964; Yairi & Ambrose, 1999). A minority of CWS go on to persist in their stutter into adulthood because around adolescence they shift from making stalling disfluencies to advancing disfluencies, possibly as a result of environmental influences such as high turn-taking pressure (Howell, Au-Yeung, & Sackin, 1999). When the plan is not ready, the speaker is cued to slow the speech rate, which they do at a young age by stalling. If the cues are repeatedly ignored, speakers make advancing disfluencies, the speech system eventually loses sensitivity to the cue to slow down and stuttering becomes persistent. Speech rate has a specific meaning in this connection insofar as it refers to rate of advancement through a message. The persistent speakers are advancing over-rapidly through the message for planning and execution to be synchronized (though conventional measures, such as syllable per minute, which would include the syllables involved in stallings would not be an appropriate index of speech rate differences).

EXPLAN is well supported by naturalistic data. The disfluencies of CWS are characterized by different linguistic properties from those of adults who stutter. In English, childhood fluency problems typically occur on FWs (Bloodstein & Gantwerk, 1967; Bloodstein & Grossman, 1981). One type of disfluency that occurs on FW is whole word repetition (Conture, 1990; Howell, 2007), as in ‘*at at at school*’. The features of childhood stuttering fit a stalling explanation, in that word repetition appears to delay a more difficult word rather than being difficult in themselves. In contrast, fluency problems for adults who stutter occur on CWs, i.e. those with semantic content. CWs are typically less frequent in English and phonologically more difficult (Dworzynski & Howell, 2004; Howell & Au-Yeung, 2007; Howell, Au-Yeung, & Sackin, 2000; Howell et al., 2006). Both these factors would make them less easily generated than FWs. Disfluencies often involve the first part of CWs (Conture, 1990; Howell, 2007), as in ‘*at ssssssschool*’, and the more phonologically difficult the word is, the more likely it is to be stuttered (Dworzynski & Howell, 2004; Howell & Au-Yeung, 2007; Howell et al., 2000, 2006). This pattern of disfluency suggests that disfluencies arise because the plan of the CW is not complete (CWs being slow to plan) and disfluencies result when the speaker nevertheless starts the word which results in part-word disfluencies which arise from this advancing process.

The differences between childhood and adulthood stuttering are consistent with the EXPLAN hypothesis that CWS produce stalling disfluencies and adults who stutter produce advancing disfluencies. This pattern is more difficult to explain in terms of the CRH because it considers disfluency across the lifespan as qualitatively the same; that is, to reflect covert repairs. Stallings have similarities with covert repairs but advancing disfluencies are qualitatively different. To explain the pattern shift in terms of the CRH, one possibility is that the change does not reflect a change in underlying mechanism, rather that CWS make covert repairs in different places from adults who stutter. However, there is no evidence for this.

Another possibility is that the change is an artifact of the cross-sectional designs that Howell and colleagues used in the past. About 80% of children, who stutter in early life, recover (Andrews & Harris, 1964), leaving a small subset who persist. The child group would reflect the disfluency characteristics of children who will later recover, as well as those who will persist. In contrast, the older age group would not include those CWS in childhood but have since recovered (Wingate, 2002). If, as the CRH suggests, children who recover from stuttering are similar to fluent speakers but those who persist constitute the ‘true’ stuttering population, then the data from the child group would reflect a mixture of ‘true’ and ‘non-true’ stuttering processes, but the adult data would reflect only the ‘true’ patterns. If these two groups differed in their speech processing, then the patterns of disfluency found across the two groups could be expected to differ. This would be in line with the CRH.

The thrust of the cross-sectional argument is negated by recent longitudinal findings (Howell, 2007). CWS at around age eight were followed up until they were 12 plus. The criteria for initial diagnosis of stuttering used clinical assessment and Riley's (1994) SSI-3. The children were reassessed at 12 plus, this time using self-report, parental report, researcher's assessment and severity based on SSI-3 again. These allowed cases where stuttering continued (persistent) to be distinguished from cases where stuttering had ceased (recovered). Patterns of stalling and advancing dysfluency were then examined. For recovered speakers, the absolute level of disfluencies decreased as they get older, but the ratio of whole FW (e.g. “I, I, I”), to advancings (e.g. “ssspilt”) remained constant. This suggests that these speakers continued to make the same proportion of stalling disfluencies, but produce fewer disfluencies of either type. Speakers whose stuttering persisted, on the other hand, showed an increased proportion of advancings, which indicates that they changed from stalling to advancing disfluencies. This is inconsistent with the CRH, which would predict that the pattern of disfluencies produced by children whose stuttering persists would be different from the start, because they differ from other children (children who recover or who are always fluent) in that they plan speech more slowly.

In summary, it is unclear whether stuttering arises because speakers with the disorder make more covert repairs, or whether they are more vulnerable to mismatches between planning and execution time and hence under pressure to overuse the advancing strategy (leading to persistence of the problem). Neither the CRH nor EXPLAN fully addresses all the empirical evidence, so both are possible explanations of childhood stuttering. This is partly because each theory was developed to account for features of naturalistic speech and neither has been extensively tested experimentally. There are arguments in favour of both naturalistic and experimental paradigms (e.g. Mowrer, 1998; Bernstein Ratner, 2000, respectively). Naturalistic data allows language to be studied in context, but makes it difficult to determine the processing demands of language at a particular locus independent of stuttering probability (Bosshardt, 1995, 2002). Experimental evidence is needed to identify causal links in a non-circular fashion. For example, in the CRH, disfluencies are explained as covert repairs but also as providing evidence for covert repairs. In EXPLAN, CWs are claimed to cause adult stuttering because they are difficult to produce, but the evidence that they are difficult to produce is that they are often disfluent (though work is taking place which indexes difficulty independently). Naturalistic data alone cannot resolve these issues, and both theories need experimental evidence from paradigms that can directly test their predictions.

Experimental psycholinguistic studies of both fluent speakers and speakers who stutter exist that use paradigms suitable for distinguishing these theories. Speech initiation time (SIT) can be used as a measure of broader timing mechanisms related to speech planning. Previous research has shown that adults who stutter tend to be slower than fluent speakers at initiating various speech-like movements, nonsense syllables, words, short phrases, and simple sentences (e.g. Adams & Hayden, 1976; Logan, 2003; Reich, Till, & Goldsmith, 1981; Watson & Alfonso, 1987).

Auditory priming studies are suitable for examining planning time and have been carried out with 3–5-year-old children. In these paradigms, an auditory sentence or syllable is presented (the prime), the child then describes a picture (the probe) and SIT is measured. SIT is shorter for both CWS and children who do not stutter (CWNS) after primes that are related to the probe. This is the case when material is primed phonologically and syntactically (Anderson & Conture, 2004; Melnick & Conture, 2003; but see Pellowski & Conture, 2005, for conflicting findings with CWS, using lexical priming). Priming can be used to reduce the planning time needed for the production of different elements in a phrase.

In the current study, the effect of priming on both the timing and fluency of speech production is investigated. Specifically, a method is introduced that selectively primes different components of a simple utterance containing two FWs followed by a CW. Selective priming of these word types aims to facilitate the planning of some parts of an utterance and not others. Though CRH does not make specific predictions concerning selective priming, it can be argued that if as suggested by the CRH, covert errors depend on the extent of phonological complexity of the target words being planned, then CWs, which are inherently phonologically more complex than FWs would show a larger priming effect. For the same reason pre-CW pausing and CW duration would be shorter. CRH would not predict any effects on CW when FW are primed or vice versa.

EXPLAN predicts a different effect of priming, on fluency and speech initiation, across words in an utterance depending on whether a CW or a FW was primed (how FW priming affects CWs and how CW priming affects FWs) which is examined here.

If EXPLAN is correct, making a CW faster to plan by priming it would reduce the number of disfluencies by reducing the chances of a mismatch occurring between planning and execution processes. This effect would be paralleled by an effect on speech timing, in that there would be less silent pausing within the speech preceding the CW, because less planning time would be needed. In contrast, making an FW easier to plan by priming it would not reduce the number of disfluencies, because the FW occurs first in the phrase and is easy to plan anyway, so the chances of a mismatch between planning and execution processes would remain the same. In this condition, the effect on speech initiation would be that more silent pausing would occur within the speech preceding the CW, because more planning time would be needed for that word. Finally, although the effects of FW and CW priming would apply to all children, if CWS would normally have slower SITs than CWNS in the absence of priming, the effect of priming the CW versus the FW would be greater for CWS than CWNS, because there would be more room for change.

The effect that priming would have on execution times is not clear. There is evidence that adults produce words with a shorter duration after they produced identical prime words (e.g. ‘sick, sick, sick’ rather than ‘sick, sit, sick, sit’) but children do not (Munson & Babel, 2005). Munson and Babel suggested that the effect of identical primes may have been manifest in the pausing intervals before word production rather than the target word durations, which would be in line with the prediction above, that priming in the current study will reduce SIT. Munson and Babel also speculated that the priming effect of identical words was masked by the production strategy of the children, who slowed down and reduced their speech intensity as they progressed through the end of the list. In the current study, the prime will be spoken only once and will sometimes include multiple words, rather than multiple repetitions of single words, so a similar slowing strategy is unlikely. It is possible that priming will influence the execution of words as well as the planning, such that primed words will have shorter durations than unprimed words. If so, this would influence fluency in the same direction as the predicted planning effect, so it would not confound results. More interestingly, it is also possible that there would be a difference between CWS and CWNS for word durations. The current study will record the word durations of both groups of children.

In summary, the hypotheses drawn from EXPLAN for the current study are as follows:

(1) CWS and CWNS will produce shorter CW durations and fewer disfluencies after CW priming than after FW priming. This effect will be greater for CWS than CWNS.
(2) CWS and CWNS will produce shorter FW durations and less silent pausing within their speech preceding the CW after CW priming than after FW priming. This effect will also be greater for CWS than CWNS.
(3) CWS will have slower SITs than CWNS.
(4) CWS will have longer word durations than CWNS.

## 2 Method

### 2.1 Participants

Twenty-four native English-speaking children participated, split into groups of CWS and CWNS. All participants were recruited from the UCL Speech Group database. The 12 CWS ranged in age from 3;10 to 8;11 (M 6;3) with 10 males and 2 females. All had a stutter diagnosed by a speech and language therapist. Age of stuttering onset was available for 10 of the CWS and ranged from 2;0 to 6;6 (M 3;6). Age of onset was not known for the other two CWS. All CWS had received Lidcombe Therapy at clinics in the London area. It was not possible to conduct an SSI-3 because secondary feature data were not available. The mean percentage of stuttered syllables in spontaneous samples was 10.87% (range 6.74–21.2%).

Data from three further CWS were collected but not included in analyses, because two (aged 9;4 and 9;3) fell outside the final age bracket of the study and one failed to comply with the procedure (not consistently repeating the prime and not responding to targets in full sentences). An upper boundary of 9 years was selected for the age group because Howell, Au-Yeung, and Sackin (1999) found that the exchange from FW to CW stuttering starts at around age 9. Including children only below 9 years means that they were likely to constitute a qualitatively homogenous group in terms of the mechanism of their stuttering. Also, the pilot experience with the two older participants indicated that the procedure was not age appropriate for them in that the stimuli were too simplistic. The 12 age and gender matched CWNS ranged in age from 3;9 to 8;9 (M 6;6), again with 10 males and two females.

### 2.2 Materials and design

‘*E-Prime*’ software was used to run the experiment on a laptop computer. The design included 20 pre-recorded auditory utterances as primes, 22 visual action-event cartoons to provide two practice items and 20 targets (created in *Microsoft PowerPoint 2000* and transferred to bitmap form for inclusion in *E-Prime*), 37 static pictures as filler items (also created in *Microsoft PowerPoint 2000* and transferred to bitmap form for inclusion in *E-Prime*). The inclusion of filler items is standard practice in a priming paradigm (e.g. Bock & Griffin, 2000; Smith & Wheeldon, 2001), to avoid a strong cumulative effect of priming by allowing time for activation to subside and to avoid a kind of ‘internal’ priming by participants being able to predict the next item. Also, the fillers had the added benefit in the current design of adding interest to the task and rendering it more ‘child-friendly’, increasing the chances that children's attention would be maintained and that they would engage with the task up to completion. A pre-recorded beep coincided with the start of the target presentation. The 20 auditory primes were pre-recorded on audiotape in a sound-treated laboratory by a 26-year-old native English-speaking adult female. Half were FW primes. These were a second person singular pronoun (matching the gender of the subsequent target cartoon) followed by the auxilliary ‘*is*’. The other half of the primes were CW primes. These were a third person present perfect verb (matching the action depicted by the subsequent target cartoon). For example, the target that depicted a boy swimming would be preceded in the FW prime condition by ‘*he is*’ whereas it would be preceded in the CW prime condition by ‘*swimming*’.

The 37 filler pictures each depicted a single animal character that the child was required to name (badger, bear, bee, butterfly, camel, cheetah, cow, crab, crocodile, deer, dinosaur, dog, dolphin, duck, elephant, fish, frog, giraffe, hedgehog, horse, kangaroo, lion, monkey, mouse, owl, penguin, pig, rabbit, rhino, sheep, snail, snake, spider, squirrel, tiger, turtle, and zebra). Table 1 contains details of the frequency of occurrence, age of acquisition and imageability of the nouns from Baayen, Piepenbrock, and Gulikers (1995), Brown (1984), Kucera and Francis (1967) and the MRC Psycholinguistic Database (1997) (no one database contains entries for all words). The two practice and 20 target action-event cartoons each depicted an intransitive action performed by a child. Half were performed by a boy and half were performed by a girl. The intransitive verbs depicted in the practice stimuli were ‘walk’ and ‘bend’. Those depicted in the 20 target stimuli were ‘cry’, ‘dance’, ‘dig’, ‘drink’, ‘eat’, ‘fly’, ‘jump’, ‘knit’, ‘paint’, ‘run’, ‘skate’, ‘skip’, ‘sleep’, ‘smile’, ‘sneeze’, ‘stamp’, ‘stretch’, ‘swim’, ‘swing’ and ‘wave’. Table 2 contains details of frequency of occurrence, age of acquisition and imageability of the verbs from Brown (1984), Kucera and Francis (1967) and Baayen et al. (1995) (again, no one database contains entries for all words). When presented, each action-event cartoon lasted 2400 ms. Each experimental item consisted of a single auditory prime that could be used to describe the subsequent target, which was repeated aloud by the child, followed immediately by a single target cartoon, which was described by the child in a single sentence. The stimuli therefore provided 20 items for analysis.

The study employed a mixed design. As a between subjects variable, half the children were CWS and half were fluent speakers. As a within subjects variable all children experienced all 20 items in both the FW and the CW conditions, presented in two blocks of 40 trials. Each block contained all 20 experimental trials (FW and CW priming), and all 37 filler trials with three randomly selected to be repeated to bring the total to 40 (animal naming),^1^ so each item was presented twice. On one presentation, each experimental item was presented with a FW prime and on the other, it was presented with a CW prime. The items were presented in pseudo-random order, with items 1–10 assigned to the FW condition and items 11–20 to the CW condition in Block 1, and the reverse assignment to conditions in Block 2, with the presentation order of trials being a randomly selected experimental trial and a randomly selected filler trial alternately. The first item in Block 1 was from the FW condition and the first in Block 2 was from the CW condition, so that the last item in the first block was never the same as the first item in the second. The blocks each lasted around 5–10 min and a short rest was permitted between them. The order of blocks was counterbalanced across participants, with half receiving Block 1 followed by Block 2 and half receiving the reverse order. The design had the advantage of controlling for how fast children recognised items by presenting every item in both conditions and measuring priming as a relative effect between the conditions.

### 2.3 Procedure

Children sat next to the experimenter, in front of a laptop computer and listened to the priming stimuli through loudspeakers. They were told that on each priming trial they would hear either a word or phrase, which they should repeat exactly. Repetition of primes is standard practice in the original production-priming paradigm (e.g. Bock's, 1986, original paradigm), to ensure that the mechanisms of speech production specifically were primed, because it is a matter of theoretical debate over whether speech production and comprehension share the same mechanisms. Though a priming effect on language production has subsequently been found to occur on the basis of comprehension stimuli, the adoption of the repetition design in the current study avoids any uncertainty over whether the mechanism that was primed is that which is involved in speech production.

After repeating the prime, children were asked to watch the cartoon that followed and describe it as soon as possible by saying ‘he is’ or ‘she is’ followed by the action. Importantly, they were told that even if they could start the sentence with the same words they had just repeated (i.e. in the priming phase), they should say the words again. They were told to respond as fast as they could whilst at the same time trying to get the answer right. This was to induce time pressure and avoid a floor effect on the dependent variables by allowing too much planning time. Children were also told that between each picture description they would see an animal and they should just name it. All participants then completed two practice trials during which they heard a prime that was unrelated to experimental trials, and then saw an unrelated target and heard a recorded example response that played after the beep. They completed the same trials immediately afterwards. Participants continued straight on to the selected 40-trial block. They were allowed a short break in between the trial blocks, if necessary.

Events on each trial were as follows. First, the screen was blank whilst a pre-recorded spoken prime was played by the computer (e.g. ‘*He is*’ for the FW condition or ‘*waving*’ for the CW condition), which the child repeated. The child's own repetition constituted the prime, as explained above. The experimenter initiated presentation of the target stimulus by pressing a key as soon as the child had finished repeating the prime sentence. Requiring the experimenter to initiate a trial allowed for variation in the time it took for the child to repeat the target and also avoided the distraction that would have been entailed if the child had pressed the key him or herself. All targets lasted exactly 2400 ms, constituting six slides, displayed for 400 ms each. A beep was produced coincident with the start of the first slide, to indicate that the target had started and mark the start of the child's response time.

The mechanism by which to present the target pictures was carefully considered and it was decided to do so manually. The authors acknowledge that there are some methodological difficulties with this, in that it introduces another potential source of error. However, there were also problems with automatic presentation, and the authors believe the manual method can be justified. Clearly, automatic presentation was not possible immediately after the target because time needed to be allowed for a child to repeat the prime. It was not possible to automatically trigger target presentation using a voice-activated microphone because multiple words were repeated, sometimes disfluently, so pausing was present. The authors considered having the primes automatically presented after a set time limit but there would be two important disadvantages of this. First, the predefined gap in which to repeat the prime would need to be set at the maximum required to avoid spoiling data from the children who took the longest. This would mean that many children would experience a gap after finishing the prime and seeing the target. This is important because the prime stimulus is the child's own production in the production-priming paradigm used here, the child's own production is the prime stimulus. A gap before this and the target could be expected to allow the priming activation to subside. Moreover, for CWS, the length of time required to articulate words can vary widely both between and within children, so the length of the gap between the prime and target would vary widely between individual children. Also, the gap would usually be longer for CWNS, who would complete the prime quicker, unless it was shorter for them, which would mean this factor would vary systematically between the groups and be potentially confounding. In sum, automatic presentation would create gaps between prime and target that would likely be confounding.

Second, slowing down the task by leaving a gap would have made the task more tedious for the children participating. In a priming design, participants need to be trying to answer quickly, or a floor effect would mask the effect. This could occur if children were not fully focussed on the task because their attention wandered whilst awaiting the target item. Also, a less interesting task would risk higher drop out rates.

In favour of manual presentation of targets is that, though there may be variation in the speed with which the experimenter pressed the button, there was no reason to expect that this would vary systematically. Therefore, across multiple trials in each condition, it would be expected that this source of error would vary randomly. The only confounding effect of this variable would be to mask a small effect size and lead to a type II error, in which case any effect that was found could be considered even more reliable. Also, the presentation of the target would be consistent relative to the individual participant, meaning that it always occurred immediately after the prime (the child's own utterance), before the predicted resulting activation had subsided.

For the reasons described, it was decided that the best solution in the current design would be for the experimenter to present targets manually. Stimulus onset asynchrony (the time period from the onset of the auditory prime to the onset of the target picture) was not constant across trials but rather the need to allow children to repeat the prime in full was prioritized.

**Fig. 1:**
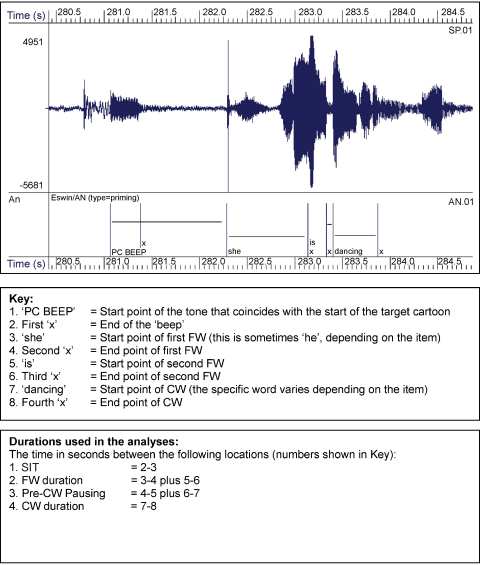
Annotated display of the responses after a child was primed with measures indicated (the key for the symbols used in the bottom line of the display and the segments used for the durations that were used in the analyses are shown at the foot of the figure). The numbers in the key refer to the perpendicular lines reading left to right. The same numbers are used to the right of the “=” sign in the section labeled “Durations used in the analyses”.

The full session for each child was recorded onto a DAT tape and later transferred to PC. Each file was analysed using the speech filing system (SFS) software developed by Huckvale, Brookes, Johnson, Pearce, Whitaker, Simpson and Breen (available as share ware at http://www.phon.ucl.ac.uk/resource/sfs/). This software allowed the user to listen to the recording, view its sound wave and create transcriptions that were time aligned to the sound wave by manually placing boundary lines on the sound wave to mark the location of significant sounds (see Fig. 1 for an annotated display of the responses after a child was primed). A time-aligned transcription was added to the sound wave to mark the starts and ends of the prime, its repetition, the beep cue, and the child's response for each trial (an example is shown in Fig. 1). The child's responses were transcribed in the Joint Speech Research Unit (JSRU) transcription alphabet and time-aligned word by word. These data were used to derive five dependent variable measures for each trial. (1) Disfluencies were counted (also broken down into FW and CW disfluencies). (2) SIT was measured by subtracting the time at which the start marker of the first response word occurred from the end marker of the beep. (3) CW duration and (4) FW duration (with 3 consisting of one word and 4 consisting of the sum of two words). Finally, (5) the amount of pausing prior to production of the CW was determined by summing the duration of silent periods that occurred between the end marker of the first response word (a FW) and the start marker of the CW (usually the final response word but on 10 occasions for CWNS and 12 occasions for CWS it was followed by another word or words, which were not included in the pausing duration^2^).

### 2.4 Classification of valid responses

Responses were classed as valid if they were a simple intransitive sentence that fitted the template: ‘Pronoun is Verb-ing’. Responses that contained additional subsequent elements (but not prior elements) were also included (see footnote 2). All other responses were excluded. For example intransitives that used nouns and not pronouns were not included because they precluded the expression of any benefits of pronominal priming.

### 2.5 Classification of disfluencies

All types of speech disfluency were included for analysis, except silent pausing, which occurs in fluent speech. This encompasses, full- and part-word repetitions, prolongations, phrase repetitions, blocks and filled pauses (e.g. ‘um’, ‘er’). Blocks were classed as disfluencies because articulatory sounds could be heard for these.

### 2.6 Reliability

To check on the reliability of the results, four files were analysed by an independent researcher. The mean differences for SIT, FW duration, pause duration and CW duration were less than 10 ms (3.4, 7.1, 5 and 9.1 ms, respectively with corresponding *sds* of 13, 14.1, 6.2 and 7.5 ms^2^).

## 3 Results

Tables 3 and 4 display the means and SDs for disfluency and timing data, respectively, for both groups in each condition. Table 5 displays the percentage of trials for both groups in each condition that contained disfluencies.

Separate ANOVAs were carried out for each of the dependent variables (disfluencies, pre-CW pausing, SIT, FW duration and CW duration).

### 3.1 Disfluency analysis

Two ANOVAs were conducted on the disfluency data, one for FW disfluencies and one for CW disfluencies. FW and CW were analysed separately as, according to EXPLAN disfluencies on the words originate from different processes. Each ANOVA used a 2 × 2 mixed design with group (CWS vs. CWNS) as a between subjects variable and prime type (CW vs. FW) as a within subjects variable.

**Fig. 2:**
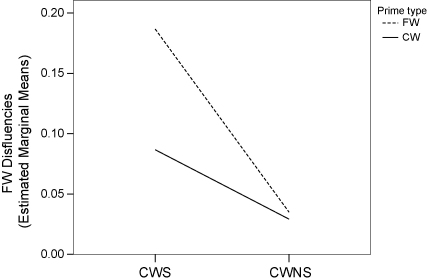
The estimated marginal mean number of FW disfluencies for each fluency group and each prime type.

For FW disfluencies, there were significant main effects of group, *F*(1, 22) = 10.947, *p* < 0.01, and prime type, *F*(1, 22) = 14.342, *p* < 0.01, and a significant interaction between the two *F*(1, 22) = 11.354, *p* < 0.01. As would be expected, CWS produced more disfluencies than CWNS. As predicted by hypothesis 1, there was a significant effect of priming in that all children produced fewer FW disfluencies after CW primes than after FW primes. Also as predicted, this difference was greater for CWS than CWNS, as shown by the interaction, which can be seen in the interaction plot of for FW disfluencies in each group and prime type (shown in Fig. 2). The figure shows the estimated marginal means, rather than observed means, because this gives a clearer picture of the interaction effect by showing the linear combination of the parameters without the error.

**Fig. 3:**
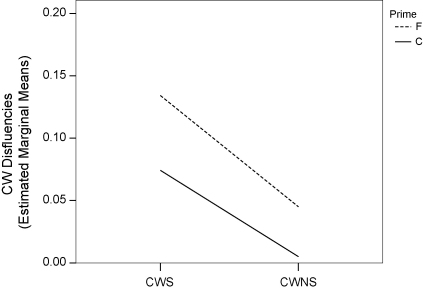
Mean number of CW disfluencies for each fluency group shown separately for function words (F) and content word (C) primes.

For CW disfluencies, there was a significant main effect of prime type *F*(1, 22) = 9.720, *p* < 0.01, but no differences between the groups (non-significant *F* value for the interaction (1, 22) = 0.389). Fewer CW disfluencies overall were produced after a CW prime than a FW prime (shown in the interaction plot of for CW disfluencies in each group and prime type, in Fig. 3). This is as predicted, except for the lack of an interaction effect.

### 3.2 Pausing

**Fig. 4:**
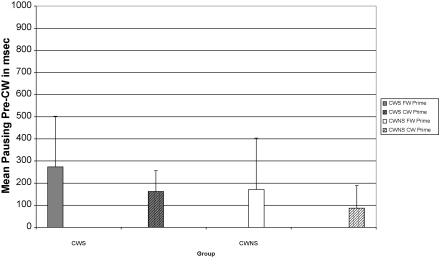
Mean length of pre-CW pausing in ms for each fluency group and each prime type.

A 2 × 2 mixed design ANOVA was performed with group (CWS vs. CWNS) as a between subjects variable and prime type the pause preceded (CW vs. FW) as a within subjects variable. For pre-CW pausing, there was a significant main effect of prime type, *F*(1, 22) = 7.885, *p* < 0.05, but no differences between the groups (non-significant *F* values (1, 22) = 1.970 and 0.148 for group and interaction, respectively) (see Fig. 4, which shows the mean length of pre-CW pausing in ms for each group and each prime type). Both groups paused for longer prior to producing the CW after a FW prime than after a CW prime. This was expected on the basis that the CW prime reduces the need for pausing to create more planning time because priming helps to ensure that the plan is complete.

### 3.3 Speech timing analysis

#### 3.3.1 SIT

For the analysis of the temporal features of participants’ speech, disfluent responses were excluded. This was because they would distort the pattern of durations by increasing durations on the word type that was stuttered in an unsystematic fashion. For example one very long disfluency on a CW could significantly increase the mean CW durations. A 2 × 2 mixed design ANOVA was performed with group (CWS vs. CWNS) as a between subjects variable, and type of prime the pause preceded (CW vs. FW) as a within subjects variable. There were no significant effects for SIT (non-significant *F* values (1, 22) = 1.175, 0.696 and 0.901 for prime type, group, and interaction, respectively).

#### 3.3.2 Duration

**Fig. 5:**
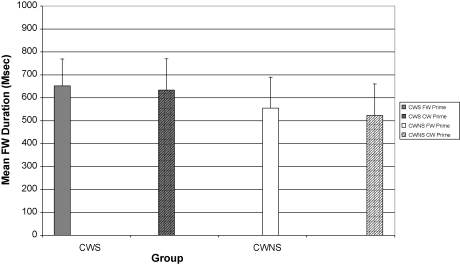
Mean function word durations, for both fluency groups and each prime type.

**Fig. 6:**
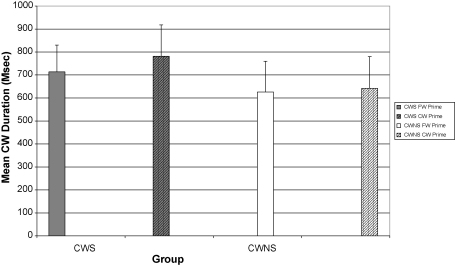
Mean content word durations, for both fluency groups and each prime type.

Again, the 2 × 2 mixed design ANOVA was used, with group (CWS vs. CWNS) as a between subjects variable, and type of prime (CW vs. FW) as a within subjects variable. One was conducted for FW duration and one for CW duration. For FW duration there were no significant effects (non-significant *F* values (1, 22) = 1.530, 3.005, and 0.104 for prime type, group, and interaction, respectively), but for CW duration, there was a significant main effect of group, *F*(1, 22) = 3.456, *p* < 0.05. CWNS produced shorter CW durations than CWS in both conditions although this was only significant when pooled across prime types (there were no effects of prime type, as shown by the non-significant *F* values (1, 22) = 3.456 and 1.289 for prime type and interaction, respectively). That is, CWS produced longer CWs, but not FWs, than the CWNS. This may reflect the fact that FW are easier to produce. These findings suggest that the CWS produced all words slower than CWNS (see Figs. 5 and 6, which show mean FW and CW durations, respectively, for both groups and each prime type).

## 4 Discussion

Three sets of hypotheses were given at the end of the introduction (concerning effects on fluency, pausing and timing behaviour). The results pertaining to each of these topics are summarised and the implications for EXPLAN and CRH are discussed.

### 4.1 Fluency

The first aspect of hypothesis 1, based on EXPLAN, was that all children (CWS and CWNS) would be more fluent when primed for CWs than for FWs because FW-priming advances when the CW-plan has to be available but not vice versa. For both CWS and CWNS, the effect on speech fluency of selectively priming different components of a target utterance is clear-cut. As predicted, both groups produced significantly fewer disfluencies after a CW prime than after a FW prime. There was no difference between incidence of disfluencies, on CWs versus FWs, although EXPLAN would predict more disfluencies on FWs at this age. It is not clear why this should be the case, although possibly some of the older children were already beginning to shift to advancing disfluencies.

The second part of the hypothesis was that the differential effect of FW and CW priming on fluency would be greater for CWS. Consistent with this, a between-groups difference was also evident: although the groups showed the same pattern, the impact of priming was significantly greater for CWS than for CWNS. These data are consistent with the EXPLAN model of speech production (Howell & Au-Yeung, 2002), but are difficult to reconcile with the CRH (Kolk & Postma, 1997), which would not predict a difference between the two priming conditions.

The findings suggest that the same process underpins the production of disfluencies for both CWS and CWNS and that it takes the form of a timing misalignment between planning and execution. The production of a CW immediately before using it in a picture description reduced the time needed to plan the CW online by activating the plan for the CW already, so that it was available in advance. This would reduce the discrepancy between the time needed to plan the CW (relatively long) and the time needed to execute the FWs (relatively short), and in turn decrease the likelihood of speaking disfluently. It appears that CWS plan CWs more slowly than do CWNS even in non-primed circumstances. The data on the temporal features of the responses support this explanation.

### 4.2 Pausing

Hypothesis 2 was that all children (CWS and CWNS) would pause more prior to producing the CW after FW primes than after CW primes, and this difference would be greater for CWS (reflecting a stalling strategy). FW priming caused both speaker groups to pause for longer before producing their target CW than did CW priming. This suggests that CW priming reduced the online planning demands for all children by supplying the CW beforehand, such that they needed a shorter pause. This explanation is consistent with the finding that all children were more fluent after CW priming. However, there was no evidence that the effect was greater for CWS than for CWNS (no main effect or interaction between fluency groups), which would be expected if CWS planned speech more slowly usually and coordinated planning and execution more poorly. Possibly, this between groups difference was obscured by the tendency for CWS to express their need for extra planning time in the form of disfluencies, rather than pausing.

### 4.3 Timing

The third hypothesis was that CWS should have slower SITs than CWNS overall, regardless of priming condition (reflecting the literature on SIT in adults who stutter) but no differences were found in SIT across fluency groups. It was not clear why this was but could reflect a cross-sectional effect, in that the adult research would be based on people whose stuttering persisted, which represents a different population from child studies.

Another interesting finding in the timing data is that the target word durations of CWS were consistently longer overall than those of CWNS, regardless of priming condition, as predicted in hypothesis 4. This suggests that in non-primed circumstances CWS would be slower to plan the CW. This would explain why CW priming increased the fluency of CWS more than that of CWNS. The slower production of CWs by CWS could be due to a planning deficit, either phonological or otherwise. Alternatively, it could be that CWS adjust to their speech problem by trying to produce difficult words (usually CWs) more slowly to avoid stuttering, either spontaneously or prompted by therapy. The duration pattern found was not predicted at the outset of the study and is not readily explained by EXPLAN. However, it could account for the difference between the groups in terms of the impact of priming on fluency, and deserves further attention.

This aspect of childhood stuttering is unlikely to be straightforward. Any future research along these lines would benefit from taking a developmental perspective that is integrated with the literature on normal phonological development in childhood. For example, there is evidence that early phonological development is influenced by infants experience of their language input, including the perception of their own productions, and the speech-production capacities with which they enter into phonological acquisition. If CWS are slower to plan language than are CWNS, then these factors could interact in complex ways over the course of development (McCune & Vihman, 2001; Vihman, 2004; Zamuner, 2003). Detailed investigation of various age groups would be necessary to unpick the origins and course of any such differences.

### 4.4 Wider discussion

The findings from the current study have implications for the nature of the process underlying a disfluent speech event. The fluency data show that fluency increases when planning time for the CW is taken out of the equation during online production. This provides the first experimental support that speech disfluency is generated by a timing misalignment at the speech–language interface, via a trade-off between the execution and planning time of different word types. However, this is not the whole story behind childhood disfluency. The current data indicates that there are other differences between the speech processing of CWS and CWNS, because the effect of priming on fluency is greater for CWS compared to CWNS, and the patterns apparent in the temporal data. This is not consistent with the EXPLAN account, which claims that before the teenage years, the difference between CWS and CWNS constitutes a difference placing along a continuum of normal fluency. The remainder of this discussion will address how this difference might best be explained.

At first glance, the current finding that CWS produce their speech slower than CWNS could be taken as support for Kolk and Postma (1997) CRH, but closer inspection of the results does not bear this out. The data are consistent with CWS having a slower planning system than CWNS, but this is not necessarily phonological in nature. The stuttering literature has provided evidence for multiple types of planning deficits to account for stuttering, including the syntactic level (e.g. Anderson & Conture, 2004; Bernstein Ratner, 1997; Karniol, 1995) and the metrical level (Wingate, 2002). Indeed, the current finding for lexical priming that showed the effect was greater for CWS than CWNS was also found by Anderson and Conture (2004) for syntactic priming. The question of which is the most relevant level of planning for fluency continues to be the subject of much debate and it would be premature to assume that the current finding reflects a phonological deficit.

A more serious problem for the CRH is that the disfluency data from the current study are better explained as the result of a timing misalignment process than as covert repairs. If all words are vulnerable to premature phonological selection by CWS, there is no reason to expect that priming one lexical class would be of less benefit than another, as was the case in this study. Also, the higher number of disfluencies found after FW priming than after CW priming applied equally to target words of both lexical classes. The CRH could explain why the provision of the CW plan would eliminate CW disfluencies, because it would remove the need to phonologically plan online. However, CRH could not explain why priming of the CW plan would reduce FW disfluencies, as it does not include any mechanism that links the planning of the two units. A trade-off scenario between the different elements of the sentence is required to explain why priming one component affects another.

Finally, the finding that FW priming leads to longer pausing before production of the target CW is consistent with the EXPLAN trade-off scenario, whereas the CRH makes no predictions concerning this. In relation to the current data, the EXPLAN provides a better explanation of the mechanisms underlying speech disfluency than does the CRH, which conflicts with the evidence. One way in which CRH could account for the results is by noting that in FW priming, the participant is presented with a sentence fragment (e.g., she is…) followed by a picture of the action to be named. These sentence fragments could lead the participant to predict the upcoming CW. Often, the predicted word will be different from the word actually presented (in the picture). If this were to be the case, FW priming in this paradigm would lead to the planning of a CW that is different from the target word. Such inadequate planning could conceivably underlie the increase in CW disfluency, after FW priming. This remains to be tested. In the remainder of this article, we consider how the EXPLAN theory can be extended to account for the slower planning of CWS compared with CWNS.

The issues at hand are helped by reflecting on the nature of the language production system. Various different types of planning appear to affect fluency but none provides a conclusive explanation on its own. This raises the question of whether a deficit that is specific to any one level of planning could adequately explain childhood disfluency. One hypothesis that can account for the stuttering literature and the current data is that stuttering reflects a problem with parallel streams of linguistic processing (e.g. Bosshardt, 1995, 2002). As an extension to the EXPLAN theory that the current data support, this could be easily integrated because both models share a focus on the central role of parallel processing in language production. There is some dual-task-based evidence from adults who stutter to support the hypothesis (Arends, Povel, & Kolk, 1988; Bosshardt, 2002), though the impact on fluency of dual-tasking varies according to how complex the secondary task is (Arends et al., 1988). In light of the developmental nature of stuttering, it would be interesting to explore the idea with children. The hypothesis is in line with current results, in that the provision of the CW plan by priming would eliminate the need to carry out parallel online linguistic processes. If the immediate cause of disfluency was the same for all speakers then all would exhibit disfluency on the most difficult part of the production process, and CW priming would help by providing the plan for the difficult word. Moreover, if CWS have more problems with parallel processing than do CWNS then priming would be even more helpful for them.

If problems with the control of parallel processes could explain childhood stuttering, one question that arises is why some children's stuttering persists into adulthood whilst most recover (Andrews et al., 1983; Yairi & Ambrose, 1999). Interestingly, an explanation can be reached by combining the parallel processing hypothesis with what is known about how children's language develops. The usage-based approach to language acquisition (Langacker, 1987) has revealed that early in development, at around 1 year of age, children do not possess knowledge of abstract linguistic relations. For example they do not correct an ungrammatical English word order used with a novel (made up) verb until around 3 years of age (Abbot-Smith, Lieven, & Tomasello, 2001). Instead, their early productions are based on item-based schemas that can be derived from the input. That is, their knowledge is a kind of mental template containing some concrete components and an abstract slot, from which they produce various instantiations like ‘I got the butter’ and ‘I got the door’ from the schema ‘I got the X’ (Lieven, Behrens, Speares, & Tomasello, 2003). There is also evidence to suggest that directly accessed, fully specified language is more easily and quickly produced than language that is accessed indirectly via abstract relations (e.g. Vogel Sosa & MacFarlane, 2002).

An interesting hypothesis is that when children are young, they can readily produce the specified parts of a schema during real-time speech, but they have relative difficulty accessing an item with which to fill the abstract slot. This could lead to an EXPLAN-like mismatch between planning and execution time as outlined in the introduction (Howell & Au-Yeung, 2002; Savage & Lieven, 2004) that would lead to disfluent speech. The hypothesis is in line with the data on childhood disfluency in that the ‘slot’ filling word would often (although not always) be a CW and the concrete items would often (although not always) be FWs. Early in development, as children were still getting to grips with using their recently acquired partially abstract linguistic knowledge, many children would be expected to exhibit disfluencies. The difference between normally developing children and those who had a parallel processing control deficit would be obscured by a ceiling effect on disfluencies created by the use of an immature language system. Later, as the system matured, the difference would be manifest as a division between recovered and persistent stuttering. This explanation could provide a developmental perspective on the parallel processing hypothesis that was formed on the basis of adult stuttering data.

Clearly, the theory outlined above is preliminary, and much empirical work would need to be done before it could be considered as anything more. It is presented as an interesting possibility, and the current data do not directly reflect on it. The current findings serve two main purposes. They offer the first direct experimental support for the EXPLAN model rather than the CRH, but they also suggest that EXPLAN is incomplete as a stand-alone explanation for developmental stuttering, particularly as concerns the difference between fluent, recovered and persistent stuttering. In future, research in this area needs to be opened up to explore additional possibilities, with an emphasis on incorporating findings from child language and non-linguistic cognitive literature.

## 5 Conclusions

The current study supports the EXPLAN model of speech production as a good account of the processes immediately underlying a disfluent speech event, although not all the predictions of EXPLAN were met and some interesting questions were raised about whether the speech systems of CWS are qualitatively different from those of CWNS. A particularly interesting area for future investigation would be to test different age groups on the current paradigm. Counter to what EXPLAN would predict, no more disfluencies on FWs than on CWs were produced by children in either group. It was suggested that this was because some of the older children were already beginning to shift to advancing disfluencies. It would be interesting to investigate developmental effects more directly by repeating the current study with children in more strictly defined age groups.

## Figures and Tables

**Table 1:** Details of the frequency of occurrence (Frequency), age of acquisition (AoA) and imageability for the nouns used in the 37 filler pictures (data from Baayen et al.,1995; Brown, 1984; Kucera & Francis, 1967; MRC psycholinguistic database 1997: No one database contains entries for all 37 words used)

Nouns	Frequency			AoA		Imagability	
	Bird	BVF	KFWF	Bird	MRC	Bird	MRC
Badger	0.8645	–	–	359	–	607	–
Bear	1.2095	9	57	220	–	601	572
Bee	1.2214	1	11	193	–	–	623
Butterfly	–	–	2	–	–	–	624
Camel	1.3994	–	1	303	–	–	561
Cheetah	−0.1739	–	1	456	–	562	–
Cow	1.6051	1	29	174	–	–	632
Crab	–	–	–	–	292	–	589
Crocodile	0.7471	–	1	316	–	–	601
Deer	–	–	13	–	281	–	624
Dinosaur	–	–	1	–	–	–	–
Dog	–	8	75	–	169	–	636
Dolphin	0.48	1	1	442	–	626	–
Duck	–	1	9	–	164	–	632
Elephant	–	–	7	–	222	–	616
Fish	1.493	–	35	275	–	578	615
Frog	–	–	1	–	258	–	617
Giraffe	0.1943	–	–	342	–	628	–
Hedgehog	0.316	–	–	366	–	639	–
Horse	2.1223	1	117	208	–	–	624
Kangaroo	0.4281	4	–	368	–	627	–
Lion	–	–	17	–	244	–	626
Monkey	1.2577	–	9	269	–	–	588
Mouse	–	2	10	–	242	–	615
Owl	–	–	2	–	269	–	595
Penguin	0.7014	–	–	392	–	620	–
Pig	–	1	8	–	233	–	635
Rabbit	–	–	11	–	206	–	611
Rhino	0.2243 (!!!)	–	–	424 (!!!)	–	591	–
Sheep	1.6033	1	23	208	–	–	596
Snail	–	1	1	–	–	–	577
Snake	–	–	44	–	289	–	627
Spider	0.8475	–	2	254	–	–	597
Squirrel	0.7885	–	1	353	–	–	642
Tiger	1.0776	–	7	331	–	–	606
Turtle	–	–	8	–	–	–	564
Zebra	0.2786	–	1	370	–	648	–

Frequencies are presented for the Bird, Franklin, and Howard (2001) analysis of Baayen et al.'s (1995) CELEX database (labeled Bird), Brown's (1984) verbal frequencies (labeled BVF), and Kucera and Francis’ (1967) written frequencies (labeled KFWF). Bird's frequencies are the logarithm of the combined written and spoken count divided by total words in the Celex database. BVF is the number of occurrences of a word per 1,000,000 spoken words and KFWF is the number of occurrences of a word per 1,000,000 written words. Age of acquisition and imageability were obtained from Bird et al. (2001) and the MRC psycholinguistic database (1997). MRC age of acquisition is from the norms of Gilhooly and Logie, multiplied by 100 to produce a range from 100 to 700 (min 125; max 697; mean 405; S.D. 120). Bird et al's (2001) AoA are ages multiplied by 100. Bird et al.'s imageability ratings are derived from a merging of the Pavio, Colorado, and Gilhooly-Logie norms: Details of merging are given in Appendix 2 of the MRC Psycholinguistic Database User Manual (Coltheart, 1981), and have values in the range 100 to 700 (min 129; max 669; mean 450; S.D. 108).

**Table 2:** Details of the frequency, age of acquisition (AoA) and imageability for the verbs used in the experiment

Verbs	Frequency			AoA		Imagability	
	Bird	BVF	KFWF	Bird	MRC	Bird	MRC
Cry	1.4681	–	48	159	–	619	478
Dance	1.566	4	90	295	–	553	510
Dig	1.6021	–	10	230	–	–	–
Drink	2.0153	25	82	166	211	573	553
Eat	2.4617	9	61	167	–	–	563
Fly	1.552	2	33	200	–	627	582
Jump	–	–	24	–	222	–	506
Knit	–	–	10	–	–	–	–
Paint	1.8561	22	37	238	–	585	567
Run	–	22	212	–	–	–	–
Skate	0.4284	–	1	357	–	562	563
Skip	0.9727	–	5	288	–	–	–
Sleep	2.11	7	65	193	–	–	530
Smile	1.9197	4	58	215	208	595	615
Sneeze	–	–	–	–	–	–	562
Stamp	1.2214	–	8	269	–	494	–
Stretch	1.8296	1	26	387	–	–	–
Swim	0.9646	1	15	275	256	612	572
Swing	1.7476	1	24	237	–	–	–
Wave	1.6572	2	46	213	–	–	542

Frequencies are presented for Bird et al.'s (2001) analysis of Baayen et al.'s (1995) CELEX database (labeled Bird), Brown's (1984) verbal frequencies (labeled BVF), and Kucera and Francis’ (1967) written frequencies (labeled KFWF). Scores are as described in Table 1.

**Table 3:** The mean and SD of number of disfluencies, for both groups (CWS, CWNS), for FW and CW prime types given separately for all disfluencies, FW disfluencies and CW disfluencies

Group	Prime type	All disfluencies (total)		FW disfluencies (total)		CW disfluencies (total)	
		Mean	S.D.	Mean	S.D.	Mean	S.D.
CWS	FW	0.32	0.21	0.19	0.13	0.13	0.17
	CW	0.16	0.19	0.09	0.10	0.07	0.17

CWNS	FW	0.11	0.08	0.04	0.04	0.05	0.04
	CW	0.05	0.05	0.03	0.04	0.01	0.02

**Table 4:** The mean and S.D. for the timing data, for both groups (CWS, CWNS), for FW and CW prime types

Group	Prime type	SIT (ms)		Pre-CW pausing (ms)		FW duration (ms)		CW duration (ms)	
		Mean	S.D.	Mean	S.D.	Mean	S.D.	Mean	S.D.
CWS	FW	984.62	459.34	273.41	228.78	652.46	134.73	713.44	116.74
	CW	993.90	311.97	162.13	93.37	633.63	123.31	780.96	136.60

CWNS	FW	814.77	213.46	171.14	232.03	555.08	211.03	625.74	134.10
	CW	954.39	370.55	86.68	102.15	522.96	136.84	643.06	137.45

The data (reading left to right) are SIT, pre-CW pausing, FW duration and CW duration.

**Table 5:** Percent trials containing one or more disfluency for each group in each prime condition

Group	Prime type	Disfluencies
CWS	FW	31.660
	CW	13.125

CWNS	FW	10.112
	CW	3.955

## Appendix A Continuing education

1. Previous work has shown that priming for children who stutter occurs for:
  a. Syntactic and lexical factors.
  b. Syntactic and phonological factors.
  c. Lexical and phonological factors.
  d. Syntactic, lexical and phonological factors.
2. Which theory or theories predict that language processing is different in children who stutter compared with children who do not stutter?
  a. CRH and EXPLAN.
  b. CRH.
  c. EXPLAN.
  d. Neither CRH nor EXPLAN.
3. EXPLAN predicts that the factors that affect stuttering are:
  a. Planning.
  b. Motor execution.
  c. Planning and motor execution.
  d. Neither.
4. The finding that, for all children, FW priming increased CW disfluency rate is predicted by:
  a. CRH alone.
  b. EXPLAN alone.
  c. Both CRH and EXPLAN.
  d. Neither.

## Footnotes

1 This design was adopted because a full 40 filler items that were depictable and recognisable could not be found, meaning three needed to be repeated.

2 On these responses, a child produced extra information after the target sentence had been successfully produced, such as ‘He is stretching’ followed by ‘high like a monkey’. Such responses were classed as valid, because they encompassed all the required elements to meet the scoring criteria. For analyses, they were included in the disfluency data in their entirety, because any influence that the prime had on planning and execution could occur at any loci in the whole utterance, so to disregard any elements of the response would be to risk disregarding data that validly reflected the effect of the independent variable (the prime type). For the timing analyses, these responses were also included, but only the basic intransitive sentence was used and the subsequent additional words were removed (‘high like a monkey’ in the example given). This was to ensure the word durations and pausing durations were done on sentences of equal length.
